# Generative FDG-PET and MRI Model of Aging and Disease Progression in Alzheimer's Disease

**DOI:** 10.1371/journal.pcbi.1002987

**Published:** 2013-04-04

**Authors:** Juergen Dukart, Ferath Kherif, Karsten Mueller, Stanislaw Adaszewski, Matthias L. Schroeter, Richard S. J. Frackowiak, Bogdan Draganski, for the Alzheimer's Disease Neuroimaging Initiative

**Affiliations:** 1 LREN, Département des Neurosciences Cliniques, CHUV, Université de Lausanne, Lausanne, Switzerland; 2 Max-Planck-Institute for Human Cognitive and Brain Sciences, Leipzig, Germany; 3 Day Clinic of Cognitive Neurology, University of Leipzig, Leipzig, Germany; 4 LIFE - Leipzig Research Center for Civilization Diseases, University of Leipzig, Leipzig, Germany; 5 Consortium for Frontotemporal Lobar Degeneration, Leipzig, Germany; Indiana University, United States of America

## Abstract

The failure of current strategies to provide an explanation for controversial findings on the pattern of pathophysiological changes in Alzheimer's Disease (AD) motivates the necessity to develop new integrative approaches based on multi-modal neuroimaging data that captures various aspects of disease pathology. Previous studies using [18F]fluorodeoxyglucose positron emission tomography (FDG-PET) and structural magnetic resonance imaging (sMRI) report controversial results about time-line, spatial extent and magnitude of glucose hypometabolism and atrophy in AD that depend on clinical and demographic characteristics of the studied populations. Here, we provide and validate at a group level a generative anatomical model of glucose hypo-metabolism and atrophy progression in AD based on FDG-PET and sMRI data of 80 patients and 79 healthy controls to describe expected age and symptom severity related changes in AD relative to a baseline provided by healthy aging. We demonstrate a high level of anatomical accuracy for both modalities yielding strongly age- and symptom-severity- dependant glucose hypometabolism in temporal, parietal and precuneal regions and a more extensive network of atrophy in hippocampal, temporal, parietal, occipital and posterior caudate regions. The model suggests greater and more consistent changes in FDG-PET compared to sMRI at earlier and the inversion of this pattern at more advanced AD stages. Our model describes, integrates and predicts characteristic patterns of AD related pathology, uncontaminated by normal age effects, derived from multi-modal data. It further provides an integrative explanation for findings suggesting a dissociation between early- and late-onset AD. The generative model offers a basis for further development of individualized biomarkers allowing accurate early diagnosis and treatment evaluation.

## Author Summary

Establishing an accurate diagnosis of Alzheimer's disease has been a major challenge in the past decades. With an increasing amount of studies aiming at detection and validation of imaging biomarkers for this disease, many apparently controversial findings have been reported over the time. The failure of current strategies to provide a consistent explanation for these differential findings motivates the necessity to develop new integrative approaches based on multi-modal data that capture various aspects of disease pathology. In our study we propose such a generative model providing a comprehensive approach towards integration of previously published differential findings in early- and late-onset AD. We believe that our analytical strategy not only provides the link between imaging biomarkers and clinical phenotype considering the effects of aging, but could also lead to new areas of research in terms of creation of new, individualized biomarkers for a more accurate diagnosis of Alzheimer's disease.

## Introduction

Neuroimaging studies using [18F]fluorodeoxyglucose positron emission tomography (FDG-PET) and structural magnetic resonance imaging (sMRI) provide substantial evidence of high sensitivity for early detection and progression assessment in Alzheimer's disease (AD) at a group and single subject level [1]–[11]. However, among these studies there are a number of discordant results in terms of spatial characteristics and magnitude of glucose hypometabolism and atrophy [12]–[14]. The supposition that age- and symptom severity-related variability are the main cause for these discrepancies has motivated researchers to adopt analytical strategies that split disease populations into subgroups depending on age- or symptom-severity (e.g. early- and late-onset AD) [12]–[14].

The question that needs to be answered is whether age and symptom severity indeed account for most of these discrepancies. In other words, what are the relative contributions of age and disease to the anatomical patterns of abnormality of structure and function? Investigation of these relationships may also provide clues to another long-time controversy – can the observed differences between young and aged AD patients be regarded as a continuum or is there a clear separation into two cohorts dependent on separate pathological mechanisms?

Recent studies have suggested that AD related brain changes may be similar to those associated with healthy aging. If so, this could explain age overestimation determined from sMR images from AD patients [15] and inaccuracies obtained with automated classification-based computer diagnostics in the eldest healthy controls and youngest AD patients [11]. However, these effects can also be explained in a more simple way by the applied methodology. Both AD and healthy aging have been linked to a decrease in grey matter (GM) volume in extensive networks covering substantial parts of the brain [16]–[18]. Partial overlaps between these networks are therefore very likely and also occur in some key regions for AD including the hippocampus and parietal cortices. A statistical classification/prediction model trained either for prediction of age or AD would therefore consider these regions as important for separation/prediction. An older healthy control would therefore have a higher probability to be misclassified as AD when applying a classifier trained on younger AD subjects. In contrast, age prediction in an AD patient using a model built on healthy controls would result in an age overestimation in this patient due to the partially overlapping network of reductions in GM volume. Both predictions are fully in line with previous findings [15], [19].

Another controversially discussed issue is the relative capability and sensitivity of FDG-PET and sMRI to detect AD related pathology. Recent studies provided evidence for the superiority of each of the two imaging modalities as compared to the other to detect AD related pathology [10], [20]. However, none of these findings can be interpreted without serious methodical considerations. Both studies restricted their analyses to univariate region-of-interest statistics to determine the power of each modality to discriminate between AD patients and control subjects. Although this approach provides a good estimation of the differential pathology in the region mostly affected by the disease, it does not at all reflect the whole pattern of AD related pathology over the whole brain. Yet, exactly this whole-brain pattern is crucial for detection and differentiation of AD from healthy aging and other neurodegenerative processes. A second methodological limitation of previous studies is related to pre-processing of FDG-PET data. These data are strongly affected by the underlying atrophy pattern. A reduction in grey matter volume in a specific region would therefore also lead to a reduction of the observed metabolic signal due to increased contribution of other tissue types. This effect is commonly known as partial volume effect (PVE) [21]. If not accounted for, this effect strongly restricts the interpretation of the observed FDG-PET signal due to a high susceptibility to the underlying atrophy. A correction for this effect is therefore necessary to enable a valid interpretation of the independent contribution of both imaging modalities to detection of AD related pathology.

To address these issues and questions we generate group level anatomical models of pathophysiological changes observed in AD using FDG-PET and sMRI data. In these models we account for PVE and integrate disease-, age- and symptom severity-associated changes in AD patients. We further dissociate them from healthy aging related changes using a combination of voxel-based general linear models (GLMs). We additionally assume that AD-induced changes are added to changes observed in healthy aging. We use the models to generate age- and symptom severity- specific whole-brain patterns of glucose hypometabolism and atrophy. To validate the obtained model and to address the questions described above, we contrapose the models' predictions in terms of anatomical plausibility to findings reported in previous studies investigating age- and AD-related changes. Thereby, we aim to provide at a group level an integrative explanation for the controversial findings described above regarding spatial characteristics and magnitude of glucose hypometabolism and atrophy in AD. We further make conclusions on the relative capability of FDG-PET and sMRI to predict AD related pathology at a group level.

We hypothesize, on the basis of the above considerations [12]–[14] that a generative model predicting age and symptom severity contributions to disease pathology based on data from two imaging modalities – FDG-PET and sMRI – would provide a robust and accurate differential pattern of glucose hypometabolism and atrophy at different ages in AD patients. We expect stronger changes in glucose metabolic compared to anatomical data in earlier disease stages, in accordance with a recently proposed model, which suggests that functional impairment precedes structural changes in AD [22]. We also hypothesize that patterns of brain atrophy associated with healthy aging would overlap those associated with disease progression yet also show a clearly distinguishable anatomical distribution pattern.

## Methods

### Subjects

To derive a generative model of age and symptom severity related changes, we extracted from the Alzheimer's disease Neuroimaging Initiative (ADNI) database (www.adni-info.org) sMRI and FDG-PET data from multiple centres of 80 patients with a clinical diagnosis of AD and 79 healthy controls (Table 1). A full list of subject and scan IDs used in this study is provided at the following location: http://www.unil.ch/webdav/site/lren/shared/Juergen/Overview_patandcon_MRIandPETdate.xlsx. For all subjects, follow-up evaluations were available for up to 5 years after initial examination. Control subjects and AD patient groups were matched for gender and age. A diagnosis of AD was based on NINCDS/ARDRA criteria [23]. Exclusion criteria for the ADNI data included the presence of any significant neurological disease other than AD, history of head trauma followed by persistent neurological deficits or structural brain abnormalities, psychotic features, agitation or behavioural problems within the previous three months or a history of alcohol or substance abuse. For most subjects, multiple follow-up FDG-PET and sMRI scans were available. Only data from the first examination date were used for analysis. The study was conducted according to the Declaration of Helsinki. Written informed consent was obtained from all participants before protocol-specific procedures were performed. Data used in the preparation of this article were obtained from the Alzheimer's Disease Neuroimaging Initiative (ADNI) database (adni.loni.ucla.edu).

**Table 1:** Subject group characteristics.

	Controls	AD	T-test (df,t,p)
Number	79	80	-
Male/Female	41/38	40/40	-
Age (years)	75.8±4.9	75.7±7.0	157,0.1,.887
Age range (years)	62–87	55–88	-
MMSE (score)	28.7±1.6	23.6±2.2	157,16.6,<.001

Mean ± standard deviation. AD Alzheimer's disease, con converters, MMSE Mini Mental State Examination, noncon non-converters.

The ADNI was launched in 2003 by the National Institute on Aging (NIA), the National Institute of Biomedical Imaging and Bioengineering (NIBIB), the Food and Drug Administration (FDA), private pharmaceutical companies and non-profit organizations, as a $60 million, 5- year public-private partnership. The primary goal of ADNI has been to test whether serial magnetic resonance imaging (MRI), positron emission tomography (PET), other biological markers, and clinical and neuropsychological assessment can be combined to measure the progression of mild cognitive impairment (MCI) and early Alzheimer's disease (AD). Determination of sensitive and specific markers of very early AD progression is intended to aid researchers and clinicians to develop new treatments and monitor their effectiveness, as well as lessen the time and cost of clinical trials. The Principal Investigator of this initiative is Michael W. Weiner, MD, VA Medical Center and University of California – San Francisco. ADNI is the result of efforts of many co-investigators from a broad range of academic institutions and private corporations, and subjects have been recruited from over 50 sites across the U.S. and Canada. The initial goal of ADNI was to recruit 800 adults, ages 55 to 90, to participate in the research, approximately 200 cognitively normal older individuals to be followed for 3 years, 400 people with MCI to be followed for 3 years and 200 people with early AD to be followed for 2 years.” For up-to-date information, see www.adni-info.org.

### sMRI data

The sMRI dataset included standard T1-weighted images obtained with different scanner types using a 3D MP-RAGE (magnetization-prepared 180 degrees radio-frequency pulses and rapid gradient-echo) sequence varying in TR and TE (repetition and echo time) with an in-plane resolution of 1.25×1.25 mm and 1.2 mm slice thickness acquired at 1.5T magnetic field strength. All raw data were pre-processed to correct for distortion and B1 non-uniformity as described on the ADNI webpage (http://www.loni.ucla.edu/ADNI/Data/ADNI_Data.shtml).

### FDG-PET data

We analysed FDG-PET data for subjects who also underwent sMRI scans. FDG-PET data were acquired with different PET-scanner types according to one of three different protocols: 1) dynamic: a 30 min six-frame acquisition (6 five-minute frames), with scanning from 30 to 60 min post FDG injection; 2) static: a single-frame, 30 min acquisition with scanning 30–60 min post injection; and 3) quantitative: a 60 min dynamic protocol consisting of 33 frames, with scanning beginning at injection and continuing for 60 min. The majority of the scans in the ADNI study were acquired with the first acquisition protocol. Images further differed in resolution, orientation, voxel and image dimensions and count statistics. The frames from 30 to 60 minutes post injection were spatially realigned to minimize inter-frame motion artefacts and a mean image of these frames was calculated for each subject. These mean images were used for further analysis.

### Image pre-processing

All data processing steps were carried out using the SPM5 software package (Statistical Parametric Mapping software: http://www.fil.ion.ucl.ac.uk/spm/) implemented in Matlab 7.7 (MathWorks Inc., Sherborn, MA). The same pre-processing algorithm was used for sMRI and FDG-PET data, as described elsewhere [11]. This procedure includes co-registration and interpolation of both FDG-PET and sMR images to an isotropic resolution of 1×1×1 mm^3^, bias correction for inhomogeneity artefacts for sMRI data, segmentation of sMRI data into different tissue classes (only the grey matter tissue class is used for further analyses), and masking of non-GM voxels in FDG-PET data. PVE correction using the modified Müller-Gärtner method [21], [24] was in the PVElab software package [25] that is compatible with SPM5 only. This procedure uses the segmented sMR images to account for PVE and for potential atrophy effects in FDG-PET. DARTEL (Diffeomorphic Anatomical Registration using Exponentiated Lie algebra) based on grey matter tissue probability maps was used for spatial normalization of data to an average size template created from all study participants [26]. Structural MR images were additionally modulated to preserve the total amount of signal from each region. The same deformation matrices used to normalise sMRI scans to a template were used to co-register the FDG-PET images. After spatial normalization anatomical regions of all subjects were located at same location in the images. Smoothing with a Gaussian kernel of 12 mm FWHM (full width at half maximum) accounted for minor misalignment errors.. FDG-PET data were intensity normalized to cerebellar mean [27] and masked to avoid big edge effects. The cerebellar region was chosen for intensity normalization of FDG-PET as it has been shown to be a region of choice for intensity normalization which is unaffected in healthy aging and early stages of AD when correcting for PVE caused by atrophy [27]–[29].

### FDG-PET and sMRI models of healthy aging and AD

**Figure 1:**
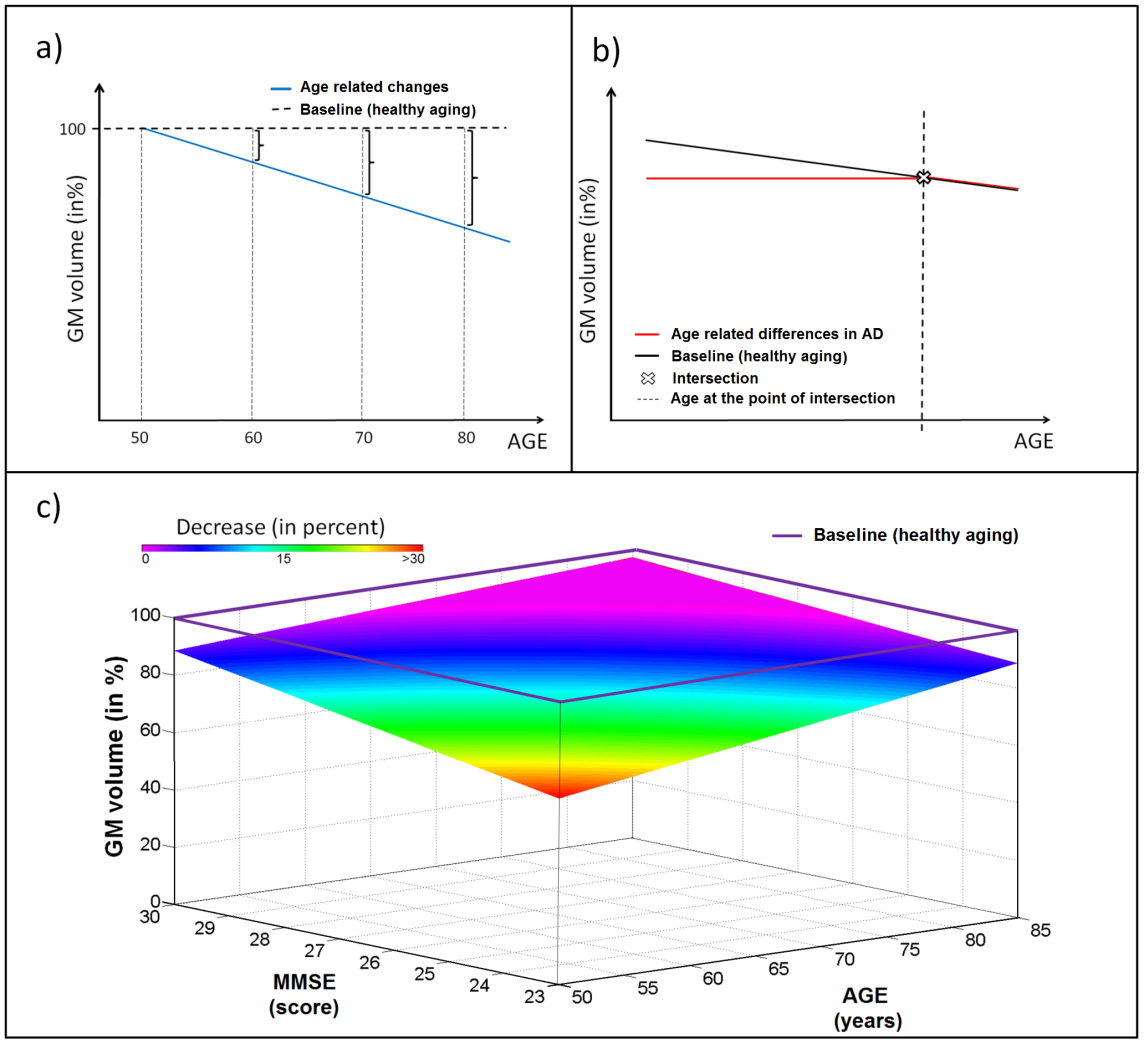
Schematic representation of voxel-wise age- and symptom severity- related models. a) Schematic representation of age-related changes in one voxel (in %) considering GM volume at the age of 50 years as baseline. b) Schematic representation of changes related to healthy aging (black line) and age-related differences in AD (red line) in one voxel. Intersection age (dotted line) represents the age at which healthy aging in this voxel becomes similar to changes observed in AD. The hinge in the red line (aging in AD) at the intersection point indicates that after the intersection age, according to our assumption of the additive impact of AD related processes to healthy aging, the healthy aging model would apply in AD patients as no pathological processes in terms of atrophy or glucose hypometabolism are longer observable after this time point. c) Decrease (in %) in GM volume observed in an exemplary voxel in AD depending on the constellation of age and symptom severity (MMSE) relative to the baseline provided by healthy aging (violet line). AD Alzheimer's disease, GM grey matter, MMSE Mini Mental State Examination.

All statistical analyses were also carried out using the SPM5 software package and Matlab 7.7. The effect of aging in healthy control subjects was estimated separately for FDG-PET and sMRI with voxel-wise linear regressions. To obtain the healthy aging component of our generative model we used the beta coefficients of aging in healthy controls to simulate voxel-wise changes in both imaging modalities for the age range 50 to 80 years (Figure 1a). The estimated values at 50 years were used as a 100% baseline. Estimated age-related changes for the whole age range for both FDG-PET and sMRI were expressed as percent decreases relative to this baseline.

To dissociate healthy aging- and AD-related changes, the variance in glucose utilization and GM atrophy explained by healthy aging was removed by voxel-wise linear regressions from all imaging data used for further models (control subjects and AD patients for both FDG-PET and sMRI; [19]). GLMs with age and symptom severity (as measured by MMSE, [30]) as regressors were built for the AD group separately for both FDG-PET and MRI. To model the interaction of both factors the product of age and symptom severity was also included. The inclusion of the interaction between age and symptom severity accounts for any differential disease progression associated with age in the AD group. In this model we took the control group mean glucose metabolism and GM volume at each voxel as a baseline. In an effort to define the specific variance attributable to AD explained by aging and symptom severity we removed variance from this baseline that was explained by these factors and their interaction (Figure 1c).

Further, we determined at what age the changes related to normal aging become similar to those found in AD. For this purpose, a third GLM, using age as the only factor, was calculated in the AD cohort for both FDG-PET and sMRI data. The ages at which the separate regressions for normal and AD associated aging intersect (referred to as intersection ages) were calculated on a voxel-wise basis (Figure 1b). To calculate these voxel-wise intersection ages 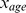 the linear regression equations for healthy aging and aging in AD (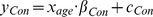 and 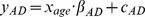, where 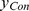 and 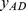 are the predicted voxel-wise grey matter volumes/glucose metabolism, 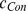 and 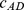 the corresponding intercepts and 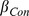 and 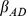 the slopes of regression lines in controls and AD respectively) were set equal and resolved for 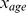. The intersection age 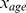 is then given by:
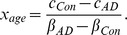
Generally, an early intersection age indicates that a region is relatively spared by AD, if healthy aging affects AD patients similarly to controls. A higher intersection age suggests that the region is predominantly affected by disease-related processes.

All three voxel-based models are based on a previously validated assumption of linearity between healthy aging- and AD symptom severity- related changes with FDG-PET and sMRI data. [16], [31]. To exclude a more complex *e.g.*, quadratic relationship between age or symptom severity in the AD cohort we calculated further regression analyses after removing variance explained by healthy aging. These comprised a) including only linear relationships for both age and symptom severity; b) additionally modelling quadratic relationships with age; and c) additionally modelling a quadratic relationship for symptom severity. Gender was included as a covariate for both imaging modalities and total intracranial volume was additionally included as a covariate for the sMRI data. A significance threshold of 0.001 uncorrected at voxel-level and 0.05 family-wise error (FWE)-corrected for non-stationarity of smoothness at cluster level was used for statistical analyses [32].

### Statistical analysis of behavioural data

Group comparisons of AD patients and control subjects for age and symptom severity were carried out using T-tests with a significance threshold of p<.05. Group differences regarding gender were evaluated using a chi-square test for independent samples. The statistical analyses were performed with SPSS 17.0 (http://www.spss.com/statistics/). A Pearson's correlation coefficient was calculated (p<.05) in the AD group to investigate the relationship between age and symptom severity.

## Results

### Subject demographic characteristics

AD patients and control subjects did not differ in age [t(157) = 0.1;p = .887]. As expected MMSE differed significantly between the groups [t(157) = 16.6;p<.001]. The comparison of AD patients and control subjects in relation to sex showed no statistical differences [χ^2^(1) = 0.06;p = .811].

### Differential pattern of changes related to healthy aging in FDG-PET and sMRI

**Figure 2:**
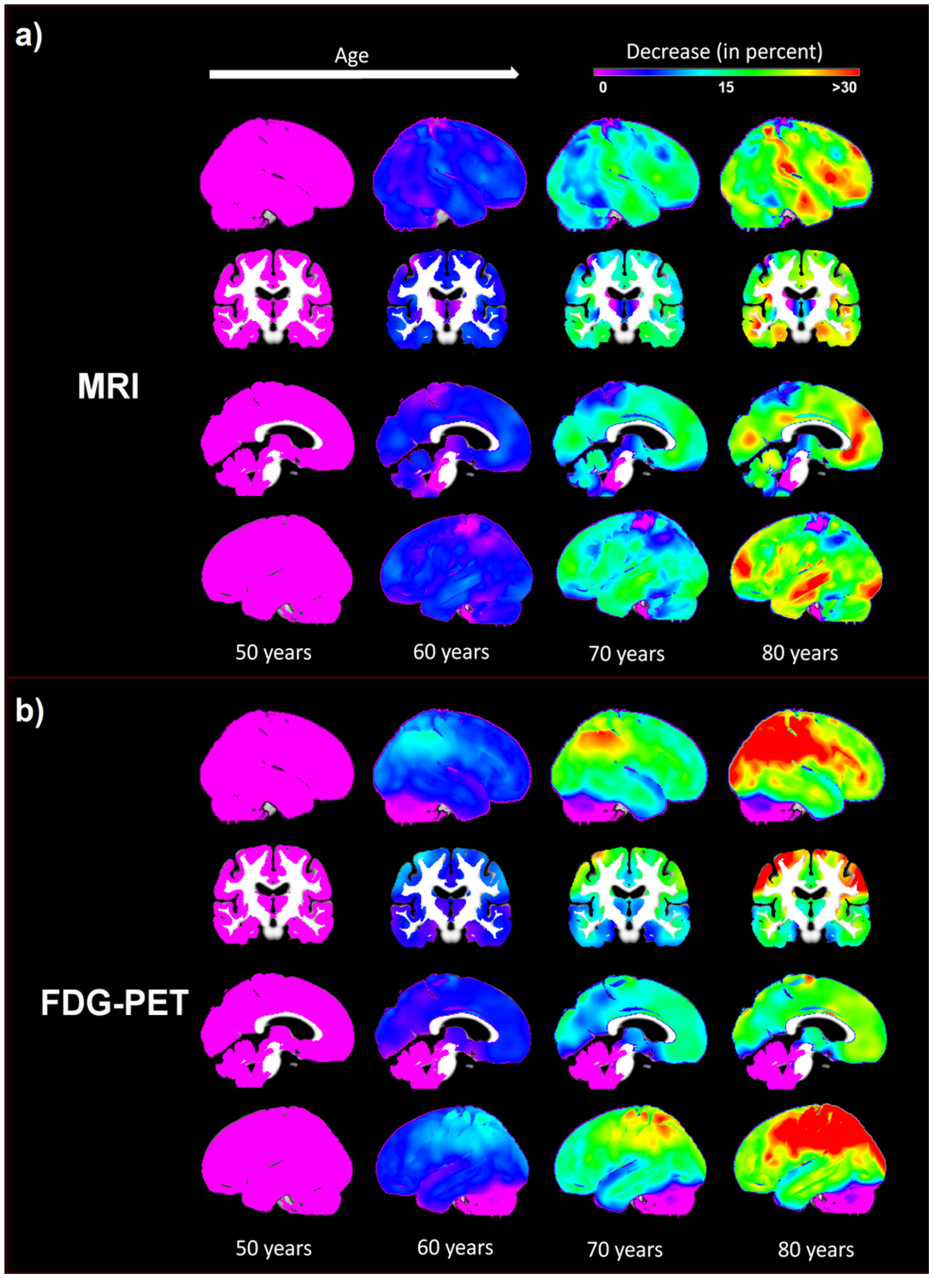
Healthy aging related changes observed in MRI (a) and FDG-PET (b) considering the expression of GM volume and glucose metabolism at the age of 50 years as baseline. FDG-PET [18F]fluorodeoxyglucose positron emission tomography, GM grey matter, MRI structural magnetic resonance imaging.

The generative model for healthy aging based on sMRI reveals a widespread pattern of grey matter volume reductions sparing only bilateral dorsal primary sensorimotor regions, brainstem, lateral thalamus and the dorsal part of caudate nucleus (Figure 2a). We observe the greatest reductions in GM volume, of more than 10% per decade, in right superior parietal lobule, superior and inferior frontal gyrus, inferior frontal sulcus, primary auditory cortex, pars triangularis, and anterior hippocampus. Left-hemispheric reductions are observed in the premotor cortex, and in superior and middle frontal gyri. Bilateral GM volume reductions are restricted to calcarine gyri, insulae, anterior cingulate cortex, superior temporal sulcus and posterior hippocampus.

The equivalent FDG-PET based model demonstrates a specific pattern of age-related metabolic changes with an age related decrease in glucose utilization of more than 10% per decade in bilateral parietal, occipital, sensorimotor, premotor, dorsolateral prefrontal and anterior insular cortices. Additionally, we see a major reduction in glucose metabolism in bilateral posterior putamina and in the left dorsal caudate nucleus (Figure 1b).

### Age- and symptom severity- related changes

**Figure 3:**
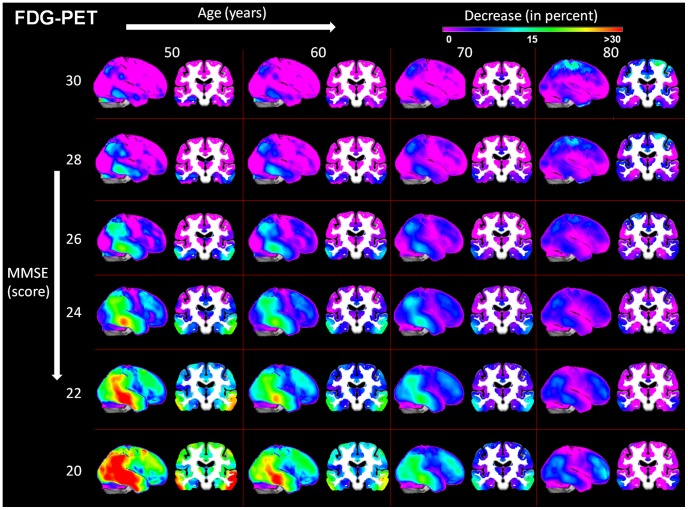
A linear model of age and MMSE related changes observed in AD in FDG-PET considering the healthy control group as baseline. AD Alzheimer's disease, FDG-PET [18F]fluorodeoxyglucose positron emission tomography, MMSE Mini Mental State Examination.

**Figure 4:**
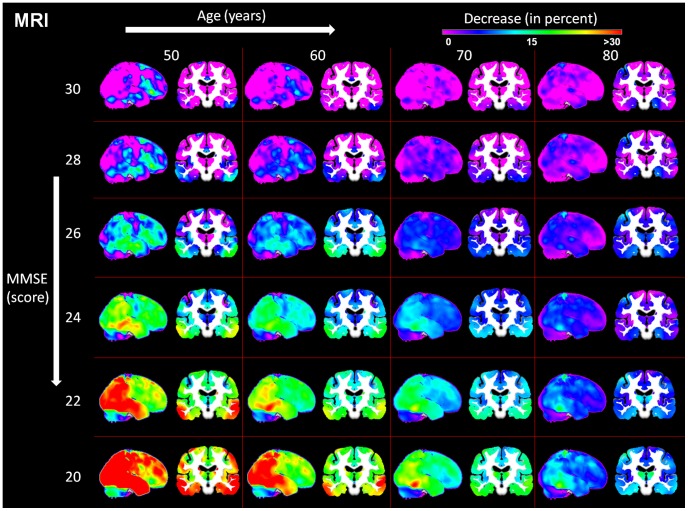
A linear model of age and MMSE related changes observed in AD in MRI considering the healthy control group as baseline. AD Alzheimer's disease, MMSE Mini Mental State Examination, MRI structural magnetic resonance imaging.

We report a negative relationship between symptom severity and both metabolism and GM volume (Figure 3) and (Figure 4). Lesser reductions in glucose utilization and GM volume are associated with greater age in AD patients.

Symptom severity related GM volume changes at age 60 years were detected throughout the brain with greatest degrees of atrophy seen in bilateral parietal, temporal, occipital, dorsolateral prefrontal, posterior cingulate and premotor cortices, the precuneus, dorsal caudate nucleus, amygdala and hippocampus. At 80 years of age, the greatest, bilateral, symptom severity related atrophy was found in parietal, temporal, occipital, primary sensorimotor and dorsolateral prefrontal cortices, the hippocampus and thalamus.

We show symptom severity- related glucose metabolism reductions bilaterally in posterior temporal, parietal, lateral occipital, dorsolateral prefrontal and premotor cortices and in the precuneus. Symptom severity related hypometabolism is less extensive at higher than lower ages.

In general, regional decreases in metabolism and grey matter volumes relative to healthy aging are significantly more pronounced in the lower compared to higher age range in AD. In the model, we observe substantial age-dependant differences in terms of hypometabolism and atrophy in AD patients compared to a healthy aging baseline even at an MMSE score of 30. At the age of 60 years these differences are bilaterally restricted to inferior frontal gyrus, premotor regions, inferior and medial temporal gyrus, cerebellum, rectal gyrus and to left parietal regions. Regions showing an initial difference in this age range do not correspond well to the anatomical pattern observed in later symptom severity stages and remain rather less affected compared to other regions. Initial differences observed at the age of 80 years are located in bilateral parietal, bilateral hippocampal and left sensorimotor regions and in both caudate nuclei.

We observe a more consistent anatomical pattern of initial differences in hypometabolism at a MMSE score of 30. For the whole age range of 60–80 years, initial glucose hypometabolism is observed in bilateral parietal, inferior temporal and posterior cingulate cortices, posterior thalamus and the precuneus. Additionally, at 80 years of age we demonstrate significant differences in bilateral primary sensorimotor and premotor regions and in the anterior temporal lobes. All regions showing initial glucose hypometabolism, except for the posterior thalamus, also show the steepest symptom severity-related metabolic decline. Regions hypometabolic only at age 80 show no specific symptom severity-related decline.

### Dissociating healthy aging from Alzheimer's disease

**Figure 5:**
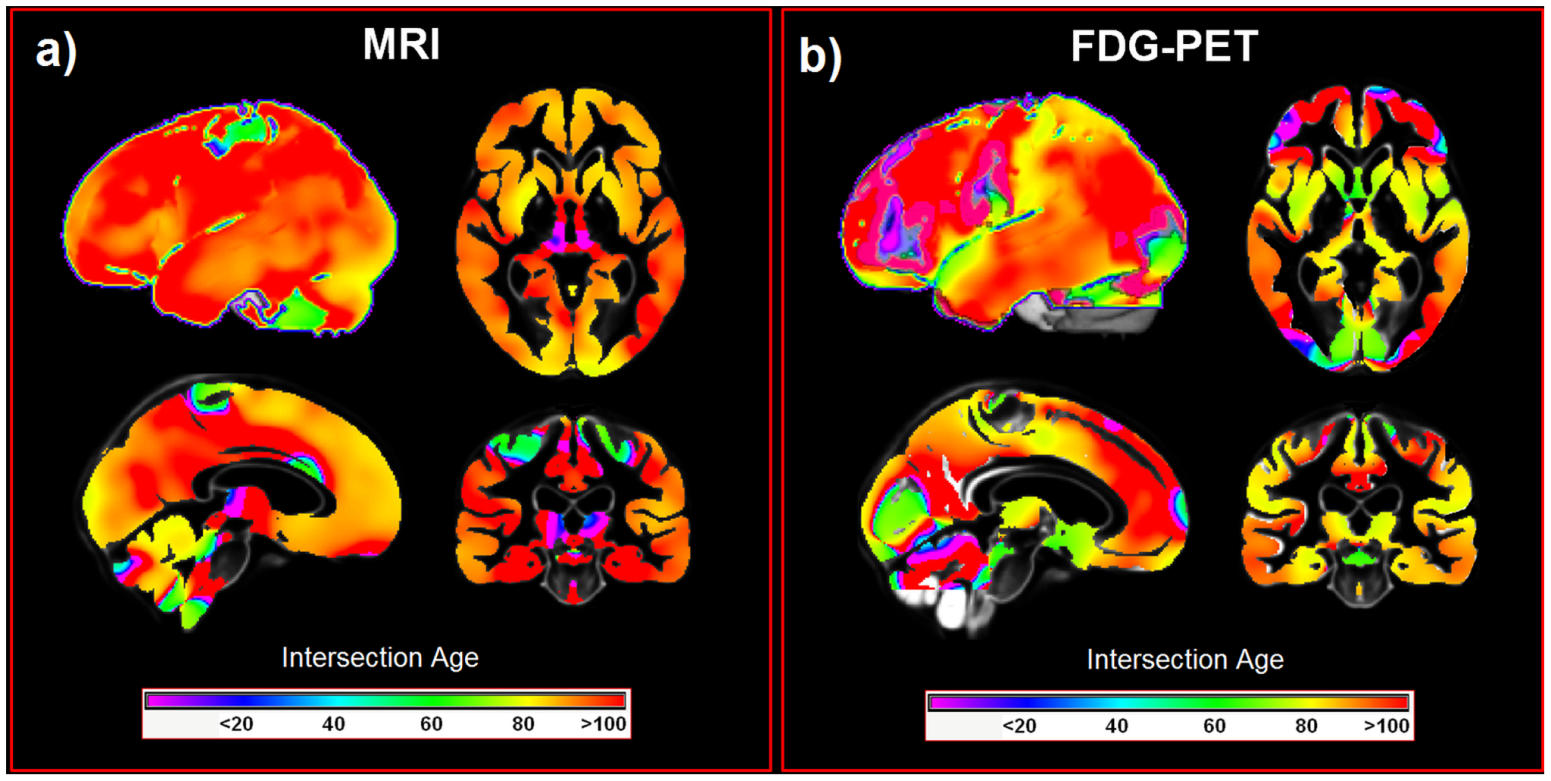
Voxel-wise intersections of healthy aging and changes observed in AD in MRI (a) and FDG-PET (b). Colour bars represent the intersection age. AD Alzheimer's disease, FDG-PET [18F]fluorodeoxyglucose positron emission tomography, MRI structural magnetic resonance imaging.

To dissociate brain changes related to healthy aging from AD pathology at specific ages we computed the intersection age of models for healthy aging and aging in AD at the voxel level (Figure 5). With sMRI, we see the highest intersection ages bilaterally in hippocampus, anterior and posterior thalamus, posterior and midcingulate, parietal, temporal, cerebellar, prefrontal and premotor regions. With FDG-PET, regions with the highest intersection ages are bilaterally restricted to precuneus, cerebellum, anterior and posterior cingulate, posterior parietal, temporal, lateral occipital, primary motor, premotor and prefrontal cortices and the left dorsal caudate nucleus.

### Linear relationship between age, symptom severity and imaging results

**Figure 6:**
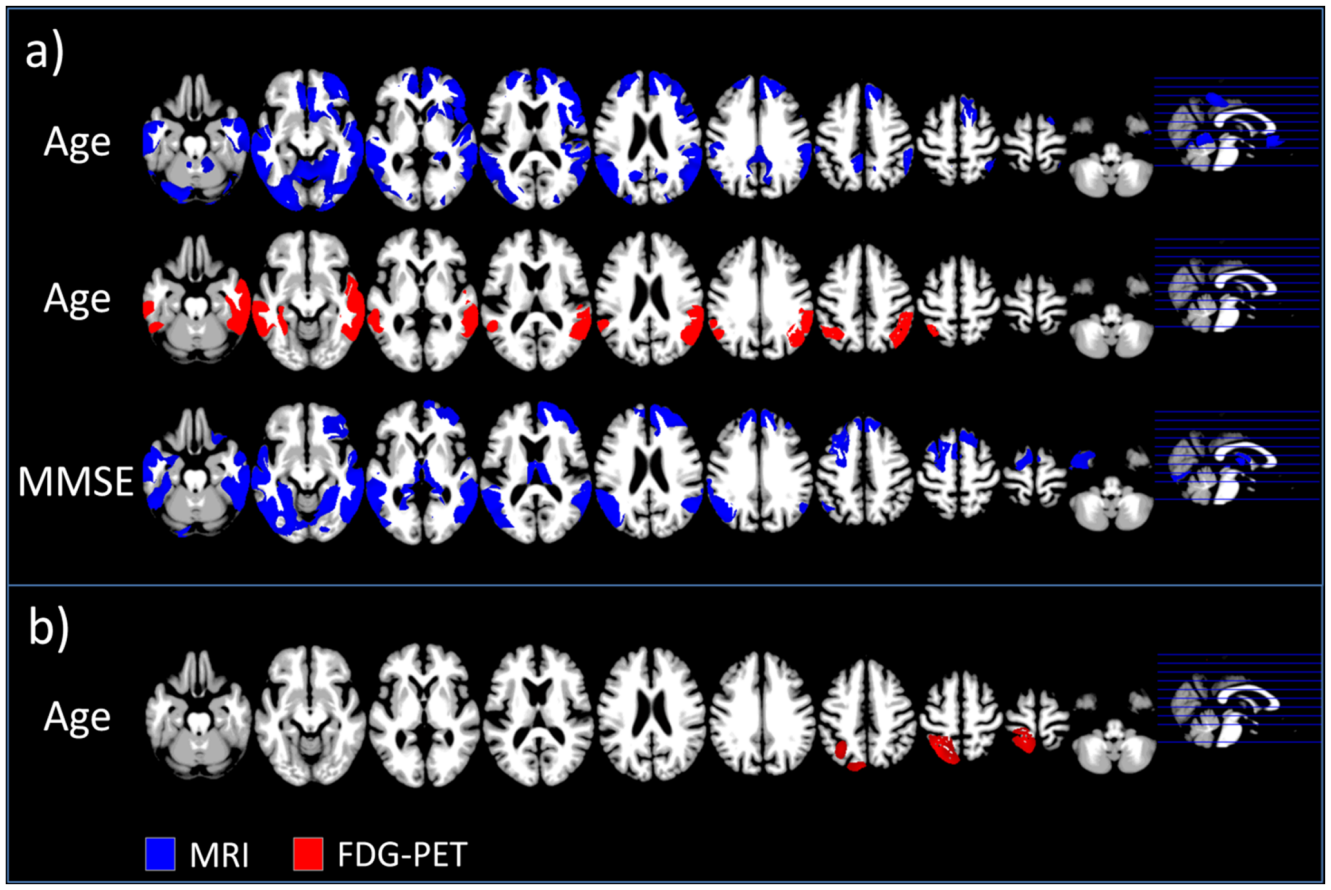
Positive linear (a) or quadratic (b) relationship observed between age and MMSE in MRI (blue) and FDG-PET (red) in AD patients after removing the variance explained by heathy aging. Only clusters are shown exceeding a significance threshold of p = 0.001 uncorrected on voxel level and p = 0.05 FWE-corrected on cluster level. AD Alzheimer's disease, FDG-PET [18F]fluorodeoxyglucose positron emission tomography, MRI structural magnetic resonance imaging.

With sMRI, the linear regression model describing the relationship between age, symptom severity and structural changes in AD showed a significant correlation for each factor with atrophy (Figure 6a). After removing the variance attributable to healthy aging, age correlates positively with GM volume in AD in right premotor and bilaterally in parietal, temporal, occipital and medial and lateral prefrontal regions. Additional significant bilateral positive correlations were observed in anterior cingulate cortex and in the cerebellum. These counter-intuitive positive correlations can be interpreted as reflecting the additional age-specific atrophy needed to produce a similar degree of symptom severity in younger compared to older AD patients. There were no significant negative correlations. Further, we found significant bilateral positive correlations with symptom severity in temporal, lateral and medial prefrontal, inferior parietal, occipital regions and in thalamus as well as the left premotor cortex. Again no negative correlations were observed.

With FDG-PET we found a significant positive correlation between age and glucose metabolism in bilateral temporal and parietal regions. There were no other significant correlations with age or symptom severity.

Inclusion of a quadratic relationship with age or MMSE into the models revealed significant positive correlations of glucose metabolism with a quadratic age coefficient only in left dorsal parietal cortex (Figure 5b). No other significant correlations were observed.

## Discussion

In this study we demonstrate that a generative model captures the anatomical and metabolic features associated with AD and healthy aging in the age range 60 to 80 years accurately and robustly. The model differentiates between the effects of aging and symptom severity in AD patients. It also provides a means to test for interactions between them, as exemplified here with disease progression and patient age. The age-dependant differential sensitivity of structural and metabolic scanning for the detection of AD pathology we demonstrate emphasises the ability of our generative model to infer expected age- and symptom severity- specific changes recorded with both imaging modalities.

Our study's three main findings support and extend observations made previously. Firstly, the spatial location of changes related to different ages and symptom severities in AD was mostly consistent with regions reported in the literature [33]–[35]. However we also identified a more widespread network of regions showing age related metabolic decreases and atrophy whilst controlling for potential PVE related changes in FDG-PET. The AD model at lower ages was substantially more extensive in terms of decreases in glucose metabolism and GM volume than at greater ages. This finding is in accordance with previous research [12]–[14].

Our second result is that in general, the magnitude of healthy aging related imaging changes between 50 and 80 years is comparable to that of changes associated with an increase in AD symptom severity at any age. Substantial regional overlap in both hypometabolism and atrophy was observed between a network of areas affected by healthy aging and that identified in AD. To interpret this overlap it is important to note that all changes related to age and symptom severity in AD we report here were calculated after removing the variance explained by healthy aging. That means that all age-related differences can be interpreted as an add-on required to normal age-related changes to induce a predefined symptom severity at the corresponding age. In previous research comparing differently aged AD groups with age-matched healthy control subjects a split of AD into different subgroups *e.g.*, early- and late-onset AD, was suggested [12]. In contrast, the results of our study indicate that the anatomically different qualitative and quantitative patterns observed in AD at different ages may be explicable by regionally inhomogeneous, age-related, baseline changes in healthy controls. This notion is supported by the observation of a similar anatomical pattern of structural and metabolic changes across the studied age range in AD patients. Overall, our results suggest a more integrative view of AD indicating that previously reported differences between early- and late-onset AD patients can be explained as an interaction of AD pathology with changes due to healthy aging. More simple, assuming that AD affects parietal and temporal regions, a strong healthy aging related decrease in glucose metabolism e.g. in the parietal cortex with at the same time relatively preserved metabolism in the temporal region would lead to an observation of both regions when comparing younger patients to younger controls. Yet, only the temporal region would be detected when comparing older patients to older controls.

A third result is in the dissociation of age- and disease- related processes inferred from the intersection ages of healthy aging and AD models. While in previous research some studies successfully applied whole-brain approaches to discriminate dementia patients from control subjects [6], [11], [36], sparse solutions based on highly discriminative features have been used in most [4], [37]–[40]. However, there is a problem with feature selection that has been neglected till now. This is the assumption of feature reliability, independent of the expression of possible confounding variables. We decided to investigate this assumption by testing whether a correlation of AD symptom severity with imaging data was also affected by the processes of normal aging as defined in a healthy population. We did this with our described method of model intersections. Our results suggest that features like local atrophy or glucose hypometabolism that discriminate between AD patients and control subjects at the age of 60 are not necessarily observed at age 80. This non-stationarity of features is mainly explained by atrophy and hypometabolism related to healthy aging, which affect some regions similarly to AD. We previously demonstrated that accounting for changes related to healthy aging improves AD detection with support vector machine classification [19]. This study extends that approach and suggests that diagnostic classifier algorithms, like machine learning techniques, when applied to the general population, need to take potential interactions of imaging features with demographic and clinical factors into account. Related to this, our intersection model also provides evidence that the pathological pattern observed in AD in some regions is clearly distinguishable from healthy aging even at very advanced age.

The obtained models could be used to improve early AD detection e.g. by training automated classifiers on age- and symptom severity- specific pathological AD patterns (features) extracted by thresholding the obtained AD model. They could also be applied directly in clinical assessment by evaluating the similarity of observed pathology in any individual to age- and symptom severity- specific patterns generated using the AD model. Thereby, to enable individual assessment, percent signal difference maps could be calculated between each subject's imaging data and imaging data generated using the healthy aging model. However, both of these approaches require careful evaluation in future studies prior to clinical application.

Nonetheless, a valid interpretation of our results needs to consider the effects of several other assumptions and limitations. First, the sensitivity of the generative model for detecting and predicting AD pathology depends on the accuracy of the model of healthy aging. The age- related, widespread patterns of brain atrophy and hypometabolism we find are consistent with previous findings [16], [31], [41]–[43]. The most prominent changes of glucose hypometabolism we observed include large parts of an occipital-parietal-frontal network. The only non-linear (quadratic) relationship between age and metabolic changes we found in an anatomically highly restricted area in the left dorsal parietal cortex. The absence of more complex relationships between these parameters indicates that linear models are very probably a sufficient approximation of the underlying structural and metabolic processes.

A major advantage of our approach is the generalizability of our model to any constellation of age and symptom severity. A further difference to conventional approaches is that the method we propose relies on a voxel-wise group mean, ignoring the variance and so allowing detection of quite minor group differences. This type of analysis, though more flexible than conventional statistics, nevertheless requires caution in the interpretation of results. Minor differences between groups of healthy subjects and AD patients could still be due to random effects unrelated to AD. However, as we model disease progression (albeit based on cross-sectional data) we would expect the magnitude of differences to increase with higher symptom severity. This assumption suggests that local differences between AD patients and control subjects that are not so correlated with it are more likely to be due to random artefacts than disease related pathology.

For all three models used in our study, we made the assumption that healthy aging affects AD patients in the same way as healthy subjects in terms of hypometabolic and atrophic changes, with AD pathology additive to changes associated with healthy aging. This assumption is in line with current views that AD as a pathological process is unrelated to healthy aging.

A further issue of interpretation of interactions is the recognised inaccuracy of clinical diagnosis of AD, which may differ in younger *vs.* older or in mildly *vs.* severely impaired patients. Any bias towards an alternative diagnosis or co-diagnosis with another dementing condition could lead to a different anatomical or hypometabolic pattern. Such a differential pattern of glucose hypometabolism was seen, for example, in sensorimotor regions only in old AD patients with low symptom severity.

Most of these problems are also common to standard statistical methods of evaluation of group differences. In group statistics one often uses a high significance threshold to avoid false positive results thus minimising their effect on between group differentiations. By contrast, our approach also provides an opportunity to evaluate the impact of possible confounding effects, such as age in this case, on the discrimination between AD patients and control subjects.

## Supporting Information

Text S1 **Acknowledgment list for ADNI publications.**
(PDF)
