# Supplementary material for: Generative FDG-PET and MRI Model of Aging and Disease Progression in Alzheimer's Disease
Source: PLoS Comput Biol. 2013 Apr 4;9(4):e1002987. doi: 10.1371/journal.pcbi.1002987 (PMC3616972; doi:10.1371/journal.pcbi.1002987)
Supplement: Text S1 — Acknowledgment list for ADNI publications. (PDF) [file pcbi.1002987.s001.pdf]

ADNI Alzheimer's Disease Neuroimaging Initiative

ACKNOWLEDGEMENT LIST FOR ADNI PUBLICATIONS

The Data and Publications Committee, in keeping with the publication policies adopted by the ADNI Steering Committee, here provide lists for standardized acknowledgement. The list consists of two parts: Infrastructure Investigators and Site Investigators. Infrastructure Investigators represent the names responsible for leadership and infrastructure. Site Investigators represent the names of individuals at each recruiting site. All papers, including methodological papers, should have an acknowledgement list that consists of Infrastructure Investigators plus the FULL list.

Part A: Leadership and Infrastructure

Principal Investigator

Michael Weiner, MD	UC San Francisco

ADCS PI and Director of Coordinating Center Clinical Core

Paul Aisen, MD	UC San Diego

Executive Committee

Michael Weiner, MD	UC San Francisco
Paul Aisen, MD	UC San Diego
Ronald Petersen, MD, PhD	Mayo Clinic, Rochester
Clifford R. Jack, Jr., MD	Mayo Clinic, Rochester
William Jagust, MD	UC Berkeley
John Q. Trojanowki, MD, PhD	U Pennsylvania
Arthur W. Toga, PhD	UCLA
Laurel Beckett, PhD	UC Davis
Robert C. Green, MD, MPH	Brigham and Women's Hospital/ Harvard Medical School
Andrew J. Saykin, PsyD	Indiana University
John Morris, MD	Washington University St. Louis

ADNI 2 Private Partner Scientific Board (PPSB) Chair

Enchi Liu, PhD	Janssen Alzheimer Immunotherapy

Data and Publication Committee (DPC)

Robert C. Green, MD, MPH	Brigham and Women's Hospital/ Harvard Medical School (Chair)

Resource Allocation Review Committee

Tom Montine, MD, PhD	University of Washington (Chair)

Clinical Core Leaders

Ronald Petersen, MD, PhD	Mayo Clinic, Rochester (Core PI)
Paul Aisen, MD	UC San Diego

Clinical Informatics and Operations

Anthony Gamst, PhD	UC San Diego
Ronald G. Thomas, PhD	UC San Diego
Michael Donohue, PhD	UC San Diego
Sarah Walter, MSc	UC San Diego
Devon Gessert	UC San Diego
Tamie Sather	UC San Diego

Biostatistics Core Leaders and Key Personnel

Laurel Beckett, PhD	UC Davis (Core PI)
Danielle Harvey, PhD	UC Davis
Anthony Gamst, PhD	UC San Diego
Michael Donohue, PhD	UC San Diego
John Kornak, PhD	UC Davis

MRI Core Leaders and Key Personnel

Clifford R. Jack, Jr., MD	Mayo Clinic, Rochester (Core PI)
Anders Dale, PhD	UC San Diego
Matthew Bernstein, PhD	Mayo Clinic, Rochester
Joel Felmlee, PhD	Mayo Clinic, Rochester
Nick Fox, MD	University of London
Paul Thompson, PhD	UCLA School of Medicine
Norbert Schuff, PhD	UCSF MRI
Gene Alexander, PhD	Banner Alzheimer's Institute
Charles DeCarli, MD	UC Davis

PET Core Leaders and Key Personnel

William Jagust, MD	UC Berkeley (Core PI)
Dan Bandy, MS, CNMT	Banner Alzheimer's Institute
Robert A. Koeppe, PhD	University of Michigan
Norm Foster, MD	University of Utah
Eric M. Reiman, MD	Banner Alzheimer's Institute
Kewei Chen, PhD	Banner Alzheimer's Institute
Chet Mathis, MD	University of Pittsburgh

Neuropathology Core Leaders

John Morris, MD	Washington University St. Louis
Nigel J. Cairns, PhD, MRCPath	Washington University St. Louis
Lisa Taylor-Reinwald, BA, HTL (ASCP)	Washington University St. Louis

Biomarkers Core Leaders and Key Personnel

J.Q. Trojanowki, MD, PhD	UPenn School of Medicine (Core PI)
Les Shaw, PhD	UPenn School of Medicine
Virginia M.Y. Lee, PhD, MBA	UPenn School of Medicine
Magdalena Korecka, PhD	UPenn School of Medicine

Informatics Core Leaders and Key Personnel

Arthur W. Toga, PhD	UCLA (Core PI)
Karen Crawford	UCLA
Scott Neu, PhD	UCLA

Genetics Core Leaders and Key Personnel

Andrew J. Saykin, PsyD	Indiana University
Tatiana M. Foroud, PhD	Indiana University
Steven Potkin, MD UC	UC Irvine
Li Shen, PhD	Indiana University

Early Project Development

Zaven Kachaturian, PhD	Khachaturian, Radebaugh & Associates (KRA), Inc<br>Alzheimer's Association's Ronald and Nancy Reagan's Research Institute
Richard Frank, MD, PhD	General Electric
Peter J. Snyder, PhD	University of Connecticut

NIA

Susan Molchan, PhD	National Institute on Aging/<br>National Institutes of Health

Part B: Investigators By Site
FULL ADNI Investigator Lists:

Oregon Health and Science University:
Jeffrey Kaye, MD
Joseph Quinn, MD
Betty Lind, BS
Sara Dolen, BS – Past Investigator

University of Southern California:
Lon S. Schneider, MD
Sonia Pawluczyk, MD
Bryan M. Spann, DO, PhD

University of California--San Diego:
James Brewer, MD, PhD
Helen Vanderswag, RN

University of Michigan:
Judith L. Heidebrink, MD, MS
Joanne L. Lord, LPN, BA, CCRC

Mayo Clinic, Rochester:
Ronald Petersen, MD, PhD
Kris Johnson, RN

Baylor College of Medicine:
Rachelle S. Doody, MD, PhD
Javier Villanueva-Meyer, MD
Munir Chowdhury, MBBS, MS

Columbia University Medical Center:
Yaakov Stern, PhD
Lawrence S. Honig, MD, PhD
Karen L. Bell, MD

Washington University, St. Louis:
John C. Morris, MD
Beau Ances, MD
Maria Carroll, RN, MSN
Sue Leon, RN, MSN
Mark A. Mintun, MD – Past Investigator
Stacy Schneider, APRN, BC, GNP – Past Investigator

University of Alabama - Birmingham:
Daniel Marson, JD, PhD
Randall Griffith, PhD, ABPP
David Clark, MD

Mount Sinai School of Medicine:
Hillel Grossman, MD
Effie Mitsis, PhD
Aliza Romirowsky, BA

Rush University Medical Center:
Leyla deToledo-Morrell, PhD
Raj C. Shah, MD

Wein Center:
Ranjan Duara, MD
Daniel Varon, MD
Peggy Roberts, CNA

Johns Hopkins University:
Marilyn Albert
Chiadi Onyike
Stephanie Kielb

New York University:
Henry Rusinek, PhD
Mony J de Leon, EdD
Lidia Glodzik, MD, PhD
Susan De Santi, PhD – Past Investigator

Duke University Medical Center:
P. Murali Doraiswamy, MD
Jeffrey R. Petrella, MD
R. Edward Coleman, MD

University of Pennsylvania:
Steven E. Arnold, MD
Jason H. Karlawish, MD
David Wolk, MD

University of Kentucky:
Charles D. Smith, MD
Greg Jicha, MD
Peter Hardy, PhD

University of Pittsburgh:
Oscar L. Lopez, MD
MaryAnn Oakley, MA
Donna M. Simpson, CRNP, MPH

University of Rochester Medical Center:
Anton P. Porsteinsson, MD
Bonnie S. Goldstein, MS, NP
Kim Martin, RN
Kelly M. Makino, BS – Past Investigator
M. Saleem Ismail, MD – Past Investigator
Connie Brand, RN – Past Investigator

University of California, Irvine:
Ruth A. Mulnard, DNSc, RN, FAAN
Gaby Thai, MD
Catherine Mc-Adams-Ortiz, MSN, RN, A/GNP

University of Texas Southwestern Medical School:
Kyle Womack, MD
Dana Mathews, MD, PhD
Mary Quiceno, MD
Ramon Diaz-Arrastia, MD, PhD – Past Investigator
Richard King, MD – Past Investigator
Myron Weiner, MD – Past Investigator
Kristen Martin-Cook, MA – Past Investigator
Michael DeVous, PhD – Past Investigator

Emory University:
Allan I. Levey, MD, PhD
James J. Lah, MD, PhD
Janet S. Cellar, DNP, PMHCNS-BC

University of Kansas, Medical Center:
Jeffrey M. Burns, MD
Heather S. Anderson, MD
Russell H. Swerdlow, MD

University of California, Los Angeles:
Liana Apostolova, MD
Po H. Lu, PsyD
George Bartzokis, MD – Past Investigator
Daniel H.S. Silverman, MD, PhD – Past Investigator

Mayo Clinic, Jacksonville:
Neill R Graff-Radford, MBBCH, FRCP (London)
Francine Parfitt, MSH, CCRC
Heather Johnson, MLS, CCRP

Indiana University:
Martin R. Farlow, MD
Ann Marie Hake, MD
Brandy R. Matthews, MD
Scott Herring, RN – Past Investigator

Yale University School of Medicine:
Christopher H. van Dyck, MD
Richard E. Carson, PhD
Martha G. MacAvoy, PhD

McGill Univ., Montreal-Jewish General Hospital:
Howard Chertkow, MD
Howard Bergman, MD
Chris Hosein, MEd

Sunnybrook Health Sciences, Ontario:
Sandra Black, MD, FRCPC
Dr Bojana Stefanovic
Curtis Caldwell, PhD

U.B.C. Clinic for AD & Related Disorders:
Ging-Yuek Robin Hsiung, MD, MHSc, FRCPC
Howard Feldman, MD, FRCPC
Benita Mudge, BS
Michele Assaly, MA – Past

Cognitive Neurology - St. Joseph's, Ontario:
Andrew Kertesz, MD
John Rogers, MD
Dick Trost, PhD

Cleveland Clinic Lou Ruvo Center for Brain Health:
Charles Bernick, MD
Donna Munic, PhD

Northwestern University:
Diana Kerwin, MD
Marek-Marsel Mesulam, MD
Kristina Lipowski, BA

Chuang-Kuo Wu, MD, PhD – Past Investigator
Nancy Johnson, PhD – Past Investigator

Premiere Research Inst (Palm Beach Neurology):
Carl Sadowsky, MD
Walter Martinez, MD
Teresa Villena, MD

Georgetown University Medical Center:
Raymond Scott Turner, MD, PhD
Kathleen Johnson, NP
Brigid Reynolds, NP

Brigham and Women's Hospital:
Reisa A. Sperling, MD
Keith A. Johnson, MD
Gad Marshall, MD
Meghan Frey – Past Investigator

Stanford University:
Jerome Yesavage, MD
Joy L. Taylor, PhD
Barton Lane, MD
Allyson Rosen, PhD – Past Investigator
Jared Tinklenberg, MD – Past Investigator

Banner Sun Health Research Institute:
Marwan Sabbagh, MD, FAAN, CCRI
Christine Belden, PsyD
Sandra Jacobson, MD

Boston University:
Neil Kowall, MD
Ronald Killiany, PhD
Andrew E. Budson, MD
Alexander Norbash, MD – Past Investigator
Patricia Lynn Johnson, BA – Past Investigator

Howard University:
Thomas O. Obisesan, MD, MPH
Saba Wolday, MSc
Salome K. Bwayo, PharmD – Past Investigator

Case Western Reserve University:
Alan Lerner, MD

Leon Hudson, MPH
Paula Ogrocki, PhD

University of California, Davis – Sacramento:
Evan Fletcher, PhD
Owen Carmichael, PhD
John Olichney, MD
Charles DeCarli, MD – Past Investigator

Neurological Care of CNY:
Smita Kittur, MD

Parkwood Hospital:
Michael Borrie, MB ChB
T-Y Lee, PhD
Dr Rob Bartha, PhD

University of Wisconsin:
Sterling Johnson, PhD
Sanjay Asthana, MD
Cynthia M. Carlsson, MD

University of California, Irvine - BIC:
Steven G. Potkin, MD
Adrian Preda, MD
Dana Nguyen, PhD

Banner Alzheimer's Institute:
Pierre Tariot, MD
Adam Fleisher, MD
Stephanie Reeder, BA

Dent Neurologic Institute:
Vernice Bates, MD
Horacio Capote, MD
Michelle Rainka, PharmD, CCRP

Ohio State University:
Douglas W. Scharre, MD
Maria Kataki, MD, PhD

Albany Medical College:
Earl A. Zimmerman, MD
Dzintra Celmins, MD
Alice D. Brown, FNP – Past Investigator

Hartford Hosp, Olin Neuropsychiatry Research Center:
Godfrey D. Pearlson, MD
Karen Blank, MD
Karen Anderson, RN

Dartmouth-Hitchcock Medical Center:
Andrew J. Saykin, PsyD
Robert B. Santulli, MD
Eben S. Schwartz, PhD

Wake Forest University Health Sciences:
Kaycee M. Sink, MD, MAS
Jeff D. Williamson, MD, MHS
Pradeep Garg, PhD
Franklin Watkins, MD – Past Investigator

Rhode Island Hospital:
Brian R. Ott, MD
Henry Querfurth, MD
Geoffrey Tremont, PhD

Butler Hospital:
Stephen Salloway, MD, MS
Paul Malloy, PhD
Stephen Correia, PhD

UC San Francisco:
Howard J. Rosen, MD
Bruce L. Miller, MD

Medical University South Carolina:
Jacobo Mintzer, MD, MBA
Crystal Flynn Longmire, PhD
Kenneth Spicer, MD, PhD

St. Joseph's Health Care:
Elizabether Finger, MD
Irina Rachinsky, MD
John Rogers, MD
Andrew Kertesz, MD – Past Investigator
Dick Drost, MD – Past Investigator
